# Penta-O-Galloyl-*β*-D-Glucose in *Pistacia integerrima* Targets AMPK-ULK1 and ERK/STAT3 Signaling Axes to Induce ROS-Independent Autophagic Cell Death in Human Lung Cancer Cells

**DOI:** 10.3389/fphar.2022.889335

**Published:** 2022-07-19

**Authors:** Acharya Balkrishna, Vallabh Prakash Mulay, Sudeep Verma, Jyotish Srivastava, Savita Lochab, Anurag Varshney

**Affiliations:** ^1^ Drug Discovery and Development Division, Patanjali Research Institute, Haridwar, India; ^2^ Department of Allied and Applied Sciences, University of Patanjali, Patanjali Yog Peeth, Haridwar, India; ^3^ Patanjali Yog Peeth (UK) Trust, Glasgow, United Kingdom; ^4^ Special Centre for Systems Medicine, Jawaharlal Nehru University, New Delhi, India

**Keywords:** penta-O-galloyl-*β*-D-glucose, autophagic cell death, UPLC/Q-TOF-MS, STAT3 inhibitor, AMPK activator, natural molecules, lung cancer, *Pistacia integerrima*

## Abstract

Natural molecules have promising perspectives as adjuvants to chemotherapies against cancer. *Pistacia chinensis* subsp. *Integerrima* (hereafter, *Pistacia integerrima*) traditionally known for medicinal values in respiratory disorders was tested for anti-lung cancer properties. The extract prepared from *Pistacia integerrima* (PI) selectively impaired the viability of lung cancer cells, A549 and NCI-H460, compared to non-cancer cells. At non-lethal concentrations, PI mitigated colony-forming, spheroid formations and metastatic properties of lung cancer cells. As a step toward identifying the phytomolecule that is imparting the anti-lung cancer properties in PI, we subjected the extract to extensive characterization through UPLC/QToF-MS and further validated the findings with UHPLC. The gallotannin, penta-O-galloyl-*β*-D-glucose (PGG), among others, was identified through UPLC/QToF-MS. PGG exhibits potential chemopreventive effects against various cancer types. However, a defined mechanism of action of PGG in restricting lung cancer progression is still unexplored. Bioactivity-guided column fractionations enabled the determination of PGG as the major phytochemical that governed PI-mediated AMPK-ULK1-dependent autophagy and apoptosis, albeit independent of intracellular ROS activation. Interestingly, the autophagy flux when inhibited restored the cell viability even in the presence of PI. The study further delineated that PI and PGG activated ERK and inhibited STAT3 to trigger apoptosis through caspase-3 and PARP 1 pathways. Collectively, the finding demonstrates that plant extract, PGG, in the PI extract effectively combats lung cancer progression through autophagic cell death by altering ERK/AMPK-ULK1/STAT3 signaling axes. The study proposes PGG as a potential AMPK activator and STAT3 inhibitor that can be exploited further in developing adjuvant chemotherapeutics against lung cancer.

## Graphical Abstract

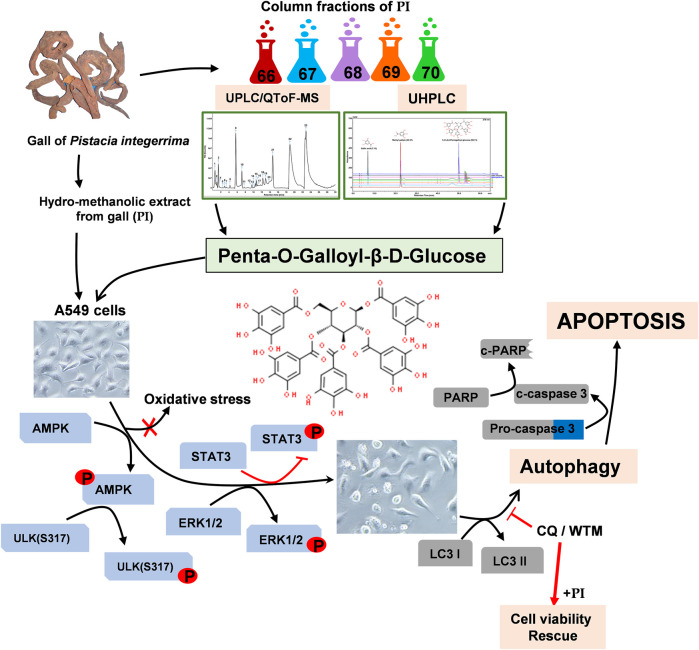

## Introduction

With an overwhelming 19.3 million new cases and 10 million deaths in 2020, cancer remains the biggest hindrance in increasing the average life expectancy globally. Lung cancer alone contributed 18% of the total cancer deaths that have made it a leading cause of cancer-associated morbidity and mortality in men and third in women (Sung et al., 2021). Classified on the basis of the origin of cell type, the epidemiological data suggest that non-small cell lung cancer (NSCLC) is a dominant contributor to around 85% of the total lung cancer cases (Inamura, 2017). Conventional therapeutics depending on the stage of the disease advocate myriad of combinatorial chemotherapy, surgery, and radiotherapy that result in long-term cancer-free survival of only 30–50% of patients with the majority encountering metastasis. Furthermore, conventional therapies are often accompanied by reported toxicities, undesirable effects, and the development of resistance to therapy. However, recently patients are being provided with rational systemic therapies against identified targets, depending on NSCLC tumor histology, the status of signature molecules, and genetic make-up (Minna et al., 2002). Unfortunately, these targeted therapies are exorbitantly expensive, with limited availability to a larger number of patients, falling into low- to middle-income groups. Hence, there is a need to develop cost-effective, alternate, or adjunct therapies that could treat the cancer pathologies and also ameliorate the adverse side effects of a conventional line of treatment.

For decades, natural products have been an important source for drug discovery and development. Traditionally plants in different forms are used to cure and heal varied diseases worldwide. Extensive documentation has listed the approved drugs from 1946 to 2019, amongst which 50% of newly approved drugs were natural small molecules or their derivatives (Newman and Cragg, 2020). Researchers are persistently reporting the novel plant molecules that exert biological activity (Greenwell and Rahman, 2015; Wen et al., 2021).

Aiming at the identification of natural molecules that can build chemotherapeutics against lung cancer, we explored the plants documented in the Indian traditional medicine system, Ayurveda. The medicinal system advocates the use of horn-shaped galls on *Pistacia chinensis* subsp. *integerrima* (J.L.Stewart) (hereafter written as, *Pistacia integerrima*) to treat asthma and other respiratory disorders (BOR, 1954). Previous studies, however, show anti-oxidant, anti-microbial, and anticancer properties in *Pistacia integerrima*, but lack an elaborate molecular mechanism, and phytomolecules that contribute to the observed therapeutic effects (BOR, 1954; Ahmad et al., 2010). The present study is an effort of exploring the anti-lung cancer potential of gall developed by an insect of the genus *Pemphigus* on the leaves and petiole of a deciduous tree, *Pistacia chinensis* subsp. *integerrima* that is commonly known as zebrawood and named *Karkatasrngakam raktapallavam* in the Sanskrit binomial system. The Pistacia tree is native to Asia and belongs to the family Anacardiaceae (Yi et al., 2008).

Our study demonstrates that non-small lung cancer cells, A549 and NCI-H460, compared to the normal non-cancer human cells, HEK293 and Beas2B, were much sensitive toward hydro-methanolic extract of *Pistacia integerrima* gall (the extract abbreviated as PI, hereafter). Interestingly, PI is distinct from other phytoextracts that exert bioactivity at higher concentrations. PI at non-lethal and sub-lethal curtails the colony formation, tumor development-like, and metastatic characteristics of A549. UHPLC followed by UPLC/QToF-MS were employed to characterize and identify the phytocompound in PI responsible for curtailing the lung cancer progression. Column fractions and their potential of targeting A549 cells suggested that PI although enriched with numerous gallotannins, the anti-lung cancer activity is by virtue of penta-O-galloyl-*β*-D-glucose (PGG). Plants, namely, *Galla Rhois*, *Rhus chinensis*, and *Paeonia suffruticosa* are known sources of PGG. Our study demonstrates the leaf gall of *Pistacia integerrima* as an unexplored natural source of PGG.

Numerous *in vitro* and *in vivo* studies have shown that PGG exhibits an array of biological properties pertaining to the anti-microbial, anti-viral, anti-diabetes, anti-adipogenic, anti-angiogenic, and anticancer properties (Lin et al., 2011; Liu et al., 2011; Pei et al., 2011; Cao et al., 2014). PGG has also been extensively studied for the identification of specific protein targets in various disease models. PGG was identified and isolated from tannic acid, one of the most potent inhibitors of human placenta aldose reductase (Sawada et al., 1989). PGG was identified in the *Moutan cortex*, and *Paeoniae radix* strongly inhibited the Na (+) and K (+) ATPase activity (Satoh et al., 1997). Other notable protein targets of PGG include salivary α-amylase, MMP9, elastase, hyaluronidase, PTP1B, and angiotensin-converting enzyme (Sawada et al., 1989; Ho et al., 2002; Gyémánt et al., 2009; Baumgartner et al., 2010; Kim et al., 2010; Chen et al., 2021). Since PGG regulates the activation and inhibition of a myriad of molecular targets several pathways concomitantly also get modulated. Signaling cascades involving caspases, TNF superfamily receptors, insulin receptors, JAK-STAT, p53, NFκB, MAPK, and ERK are well-studied examples that are revamped with PGG (Zhang et al., 2009; Cao et al., 2014). Among different cancer types, PGG has been shown to be effective against prostate cancer, breast cancer, melanoma, leukemia, and liver cancer either by inhibiting the cell cycle or by inducing apoptosis. Additional studies that show anti-angiogenic and anti-metastatic reports further establish that PGG is indeed an anticancer molecule (Zhang et al., 2009). However, in lung cancer, only the anti-angiogenic effects of PGG *via* COX2 and VEGF have been studied (Huh et al., 2005). Hence, our study aims to define the mechanism of action employed by PGG to inhibit lung cancer progression.

Our study demonstrates that PGG in PI-induced autophagy *via* the AMPK pathway that further diverts cells towards apoptosis. Interestingly, the autophagic cell death through PGG and PI is independent of ROS. PI through PGG impedes STAT3 activation but activates ERK signaling, which plays a central role in *en routing* lung cancer cells toward apoptosis. Our study reveals a natural source of PGG and an underlying molecular mechanism that can be further exploited to develop adjuvant chemotherapy against lung cancer.

## Materials and Methods

### Chemicals

Cell culture media, DMEM (Dulbecco’s modified Eagle’s medium) and RPMI 1640 (Roswell Park Memorial Institute-1640), antibiotic-antimycotic (100X), Dulbecco’s phosphate buffered saline were purchased from Gibco, United States. Fetal bovine serum (FBS) and 0.5% trypsin-EDTA were purchased from Hi-media Laboratories Pvt Ltd., India. Chemicals, hydroxychloroquine sulfate (H1306) and acridine orange 97412), were purchased from TCI Chemicals Pvt Ltd., India; crystal violet was obtained from Loba Chemie, India. Wortmannin (J63983) was procured from Alfa Aesar, United States, and penta-O-*β*-D-glucose hydrate (G7548, 10 μM equivalent to 9.4 μg/ml), monodansylcadaverine (D4008, dansylcadaverine), DCFDA (D6883), DAPI (D9542), and 2 mM L-glutamine (G7513) were purchased from Sigma-Aldrich, United States. The AR-grade solvents, toluene, ethyl acetate, formic acid, acetic acid, and methanol (HPLC grade), were procured from Merck India Pvt. Ltd. Acetonitrile from Honeywell (34967, Germany) and deionized water obtained from a Milli Q system (Millipore, United States) were utilized for the experiments. Gallic acid (91215, Sigma-Aldrich, United States) and methyl gallate (G0017, TCI Chemicals Pvt. Ltd., India) were used as authentic standards in UHPLC.

### Plant Procurement, Hydro-Methanolic Extract Preparation, and Column Chromatography

Galls present on *Pistacia chinensis* subsp. *integerrima* (J.L.Stewart) Rech. f. [Anacardiaceae] (hereafter in this study, *Pistacia integerrima*) were collected, compared, and authenticated with the Patanjali Research Foundation Herbarium (collection no. 234, accession no. 1590) at Haridwar, Uttarakhand, India (Yi et al., 2008).

The plant material weighing 203 g was cleaned, pulverized, and extracted in 2 L of water: methanol (1:1) solvent at 60–65°C for 2 h under reflux conditions. The extract was filtered through a 10-μm filter cloth, and the extract that remained in the cloth was again subjected to two more rounds of extraction repeats maintaining all the processes described previously. Finally, the filtrate was pooled and concentrated in a rotary evaporator under reduced pressure. The process yielded a 64.5% (w/w) light brown colored hydro-methanolic extract powder. We prepared 100 mg/ml of extract in 30% DMSO in autoclaved, 0.2-μm filter-sterilized water. For treatments, the extract was further diluted in cell culture media.

For column chromatography, 2 g of hydro-methanolic extract was adsorbed on silica loaded in a silica-containing column. The elution was initiated with chloroform followed by methanol in varying ratio combinations to increase polarity. Simultaneously, a TLC system (chloroform/methanol/water/acetic acid: 7.5/2/0.25/0.25 v/v) was developed for tracking the progress of the elution. A total of 34 aliquots were collected and pooled into 5 fractions on the basis of similarities in their TLC pattern. Fractions were concentrated to dry crystals and further analyzed by UPLC/QToF-MS and UHPLC.

### Cell Culture Maintenance

The human non-small cell lung cancer cell lines, A549 and NCI-H460; human bronchial epithelial cells of non-cancerous origin, Beas2B; and human embryonic kidney cells, HEK293, were obtained from ATCC-licensed cell repository at the National Centre for Cell Science (NCCS, Pune, India). A549 and HEK293 cells were propagated in DMEM (Dulbecco’s modified Eagle medium) supplemented with 10% fetal bovine serum (FBS), 1% anti-anti (antibiotic antimycotic, Gibco). NCI-H460 and Beas2B were propagated in RPMI 1640 supplemented with 10% fetal bovine serum (FBS), 2 mM L-glutamine, 1% anti-anti (antibiotic antimycotic, Gibco). The growing conditions were maintained at 37°C and 5% CO_2_ in a humified incubator. Cryopreserved cells were passaged at least twice before using for the experiments in this study.

### Cell Viability, Cell Cycle, and Apoptosis

Cells were seeded at 0.8-1 × 10^4^/ml per well in 96-well tissue culture plates. Post 24 h, 10% FBS-containing medium was replaced with 2% FBS-supplemented media cells and different concentrations of PGG or PI extract (0.1, 0.3, 1, 3, 10, 30, and 100 μg/ml) for the indicated time. Hydroxychloroquine sulfate (CQ) at 10 μM and wortmannin (WTM) at 500 nM were treated before 1 h of PI treatment in cells. Three hours before the indicated time period, Alamar blue at a working concentration of 15 μg/ml was added to the respective media. The fluorescence units (FU) were recorded in an EnVision multi-plate reader (PerkinElmer, Waltham, MA, United States) at excitation and emission wavelengths of 560 and 590 nm, respectively. Percent cell viability is calculated using the following formula:

% Cell viability = [(FU _Treated_- Blank)/(FU _Untreated_- Blank)] × 100.

The half-maximal inhibitory concentration was determined in GraphPad Prism 8.0 by plotting a non-linear dose–response curve.

For apoptosis evaluation, cells were stained with FITC Annexin V-Propidium iodide (PI) according to the manufacturer’s protocol (Invitrogen). Cells were acquired in a Attune acoustic focusing flow cytometer (Thermo Fisher, United States) and analyzed in FCS Express software version 7 (*De Novo* Software, Los Angeles, CA).

### Clonogenic Assay, Spheroid Formation, and Migration Assay

A549 cells, seeded at a density of 1 × 10^4^ cells per well of six-well tissue culture plates (cell counter, Bio-Rad), were treated with PI extract at a concentration of 0.1, 0.3, 1, 3, 10, and 30 μg/ml for 72 h time period. Alternatively, A549 cells were pre-treated with PI at 0.1, 0.3, and 1, 3 μg/ml for 72 h, and then re-seeding was performed for clonogenic assay, spheroid formation, and migration assay. For the clonogenic assay, 1 × 10^3^ cells per well in a six-well tissue culture plate were seeded and incubated in a humidified CO_2_ incubator at 37°C for 10 days with half replacement of culture media after every 3 days. Cells in each well were fixed with 100% methanol and stained with 0.2% crystal violet. Wells were thoroughly washed with water, air-dried, and imaged using a Canon camera. Colonies were counted to calculate plating efficiency and survival fraction as per the following formula, described previously (Franken et al., 2006):

Plating efficiency = (number of colonies formed/number of cells seeded) × 100;

Survival fraction = plating efficiency _Treated_/plating efficiency _Untreated_.

For spheroid formation, the pre-treated cells were resuspended to attain a density of 1 × 10^6^/ml. A measure of 20 μL of this cell suspension was dropped on low-attachment plates to allow A549 cells to grow into spheroid through the hanging drop method under normal growing conditions. Post 72 h, A549 spheroids were imaged under a brightfield microscope.

For transwell migration assay, 100 μL of 1 × 10^6^/ml cell density in 0.1% bovine serum albumin containing serum-free media was plated in the inner chamber of the transwell insert (8 μm pore size, Costar, Corning, 6.5-mm-diameter inserts, 24-well plates). A volume of 600 μL of 10% FBS-containing media was added to the lower chamber of the transwell plate, whereas wells serving as negative control received serum-free media only. After 6 h of incubation under normal growing conditions, the cells toward the inner side of the insert were removed using a cotton swab, while the ones toward the bottom were fixed in methanol and stained in 0.2% crystal violet. Cells were imaged and counted under the brightfield microscope (Olympus BX43, MANTRA, Perkin Elmer, United States) to determine the number of migrated cells that were pre-treated with respect to the untreated. Since the stock of PI extract was prepared in 30% DMSO, the vehicle wherever indicated received 0.09% DMSO corresponding to a 30 μg/ml dose of PI.

### Monodansylcadaverine Staining

To assess the increase in autophagic vacuoles, A549 cells post-treated with either PI alone or in the presence of CQ at 10 μM were stained with 50 μM MDC in PBS for 10 min at 37°C. Cells were washed thrice with PBS and lysed in 10 mM Tris–HCl, pH 8 containing 0.1% Triton X-100. Fluorescent units of intracellular MDC were recorded using an EnVision multimodal plate reader (PerkinElmer, United States) at excitation and emission wavelengths of 380 and 525 nm, respectively. To normalize the measurements to the number of cells present in each well, a solution of ethidium bromide was added to a final concentration of 0.2 mM, and the DNA fluorescence was measured (excitation wavelength 530 nm and emission filter 590 nm). Fluorescence units corresponding to MDC of each well were normalized with respect to fluorescent units of ethidium bromide. Fold change in MDC staining was calculated by dividing normalized fluorescent units of treated by untreated (Munafó and Colombo, 2001). For starvation, cells were washed and incubated with Hank’s Balanced Salt Solution (HBSS, Gibco, United States) for 6 h.

### Acridine Orange Staining

Acridine orange staining was performed to determine the autophagy levels by staining acidic vesicular organelles (AOs). A549 cells, post-treated with PI or starvation for 6 h in Hank’s Balanced Salt Solution (HBSS, Gibco, United States), were washed with PBS and incubated with 1 μg/ml of acridine orange in PBS at 37°C for 5 min. Cells were imaged under a fluorescent microscope (Olympus BX43, MANTRA, Perkin Elmer, United States) after staining.

### Intracellular ROS Determination

To detect the intracellular ROS levels in A549 cells, 24 h post PI treatment, cells were incubated with 10 μM of fluorescent probe 2′,7′-dichlorodihydrofluorescein diacetate (H_2_DCF-DA, D6883, Sigma, Sigma, Hertfordshire, UK) in HBSS (Gibco, United States) for 40 min. Absolute fluorescent units were recorded using an EnVision multi-plate reader (PerkinElmer, Waltham, MA, United States) at an excitation/emission wavelength of 485/535 nm. Fluorescent units were expressed into fold change in ROS with respect to untreated. A549 cells treated with 100 μM H_2_O_2_ were considered a positive control for the experiment. Under similar experimental conditions, DCFDA fluorescent units expressed as percent max count were also evaluated through flow cytometry (Attune acoustic focusing flow cytometer, Thermo Fisher, United States).

### Preparation of Cell Lysates and Western Blotting

A549 cells were treated as indicated followed by lysis in cold RIPA buffer (50 mM Tris, pH 8.0, 150 mM NaCl, 1.0% NP-40, 0.5% sodium deoxycholate, 0.1% SDS, 1 mM EDTA, freshly supplemented with PhosSTOP (04906845001, Roche, Germany), and complete protease inhibitor (A32963, Thermo scientific, United States)). The lysates were resolved in 10 or 12% SDS-PAGE followed by transfer to a 0.2-μm PVDF membrane (1620177, Bio-Rad, Hercules, CA). Antibody dilutions, incubation time, and temperature conditions were followed according to the manufacturer’s protocol. Blots were developed using chemiluminescent HRP substrate (ECL, Millipore) in an ImageQuant LAS 500 (GE Healthcare, United States) instrument for chemiluminescence detection. Immunoblots were further processed using ImageQuant TL software provided with the instrument. Primary antibodies against cleaved PARP 1 (44-698G), cleaved caspase 3 (PA5-114687), LC3A/LC3B (PA1-16931), phospho-STAT3 (Tyr 705; MA5-15193), STAT3 (MA1-13042), AMPKα (MA5-15815), phospho-ERK1/2 (Thr 202, Tyr 204; 36-8800), and actin (MA5-11869) were procured from Invitrogen, United States. Phospho-AMPKα (Thr 172; 2535S), phospho-ULK1 (Ser 317; 37762), and phospho-ULK1 (Ser 757; 14202) were purchased from Cell Signaling Technology (Danvers, MA). All primary antibodies were used at 1:1000-1:2000 dilution. Anti-rabbit and anti-mouse HRP tagged secondary antibodies (Invitrogen) were used at dilutions of 1:5000–1:10,000.

### Ultra-Performance Liquid Chromatography/Quadrupole Time-of-Flight Mass Spectrometry (UPLC/QToF-MS)

Solutions of PI extracts at 4 mg/ml concentration and fraction numbers 66, 67, 68, 69, and 70 at 2 mg/ml concentration were prepared in methanol. The solution was sonicated and passed through a 0.2-μm filter before analyzing it on Xevo G2-XS QToF with Acquity UPLC-I Class in the negative mode of ionization with UNIFI software (Waters Corporation, Milford, MA, United States). The mass spectrometry with electrospray ionization (ESI) operated in MS^E^ mode in a mass range of 80–1200 m/z. Other parameters include the acquisition time of 30 min, low and collision energy set at 6 eV and 15–50 eV (ramp), respectively with a cone voltage of 40 V, and capillary voltage of 2 kV. Source temperature of 120°C and desolvation temperature of 500°C, cone gas flow at 50 L/h, and desolvation gas flow at 900 L/h were the other internal parameters optimized for PI extract and fractions. Mass accuracy was maintained using 0.2 ng/ml of external reference (lock spray with leucine enkephalin) infused at a flow rate of 10 μL/min to generate a reference ion for the negative ion mode [(M - H)^-^ m/z 554.2620]. The scan time for lock spray was assigned as 0.5 s with an interval of 30 s. The separation was carried out using the Nucleodur C18 Gravity column (100 × 2.0 mm, 1.8 µm) maintained at 35°C. The elution was carried out at a flow rate of 0.3 ml/min using gradient elution of mobile phase 0.1% formic acid in water (mobile phase A) and 0.1% formic acid in acetonitrile (mobile phase B). The volume ratio of solvent B was changed as follows: 5% B for 0–3 min, 5–15% B for 3–10 min, 15% B for 10–20 min, 15–60% B for 20–30 min, 60–5% B for 30–31 min, and 5% B for 31–35 min. A volume of 1 μL of the prepared solution of PI extract and fractions was injected for the screening, and the chromatograph was recorded for 30 min. Compounds were analyzed by their respective mass-to-charge ratio and fragmentation patterns. The mass/charge (m/z) ratio was selected based on the molecular ions of these compounds (Balkrishna et al., 2021a). Data acquisitions were collected under the negative mode of ionization using full-spectrum scan analysis.

### Ultra-High-Performance Liquid Chromatography (UHPLC) Analysis

Standards of gallic acid, methyl gallate, and penta-O-*β*-D-glucose hydrate (PGG) were dissolved in methanol to prepare the 100 ppm of the working standard, and 2.9, 6.0, 6.8, 5.9, 6.3, and 6.2 mg of fraction numbers 66, 67, 68, 69, 70, and sample PI extract, respectively, were dissolved in 10 ml of methanol for use in HPLC analysis. The quantification of gallic acid, methyl gallate, and PGG in PI extract and fractions was performed by UHPLC (Shimadzu Prominence XR, Japan) armed with a quaternary pump (Nexera XR LC-20AD XR) comprising a degassing unit (DGU-20A 5R), DAD detector (SPD-M20 A), and auto-sampler (Nexera XR SIL-20 AC XR). Separation was achieved using a Shodex C18-4E (5 μm, 4.6*250 mm) column subjected to binary gradient elution. The two solvents used for the analysis consisted of 0.1% glacial acetic acid in water (solvent A) and 0.1% glacial acetic acid in acetonitrile (solvent B). Gradient programming of the solvent system was initially at 5% B 0–5 min, 5–12% B from 5 to 25 min, 12–17% B from 25 to 35 min, 17% B from 35 to 45 min, 17–25% B from 45 to 50 min, 25–40% B from 50 to 59 min, 40–5% B from 59 to 60 min, and 5% B from 60 to 65 with a flow rate of 1.0 ml/min. A volume 10 µL of standard and test solution were injected, and the column temperature was maintained at 35°C. The wavelength was set at 278 nm (Balkrishna et al., 2021b).

### Data Analysis

Software GraphPad Prism 8.0 and MS Office Excel 2010 were used to execute statistical calculations. Data sets of each group are expressed as mean ± standard error of the mean (SEM) unless indicated. *p*-values for the data sets were considered significant if *p* < 0.05 (*p < 0.05, **p < 0.005, ***p < 0.0005, #*p* < 0.00001) and not significant (ns) if *p* > 0.05. To determine the *p*-values, analysis of the mean values was performed through one-way or two-way analysis of variance (ANOVA) with Dunnett’s multiple comparison test.

## Results

### *Pistacia integerrima* Induces Selective Toxicity in Non-Small Cell Lung Cancer Cells

**FIGURE 1:**
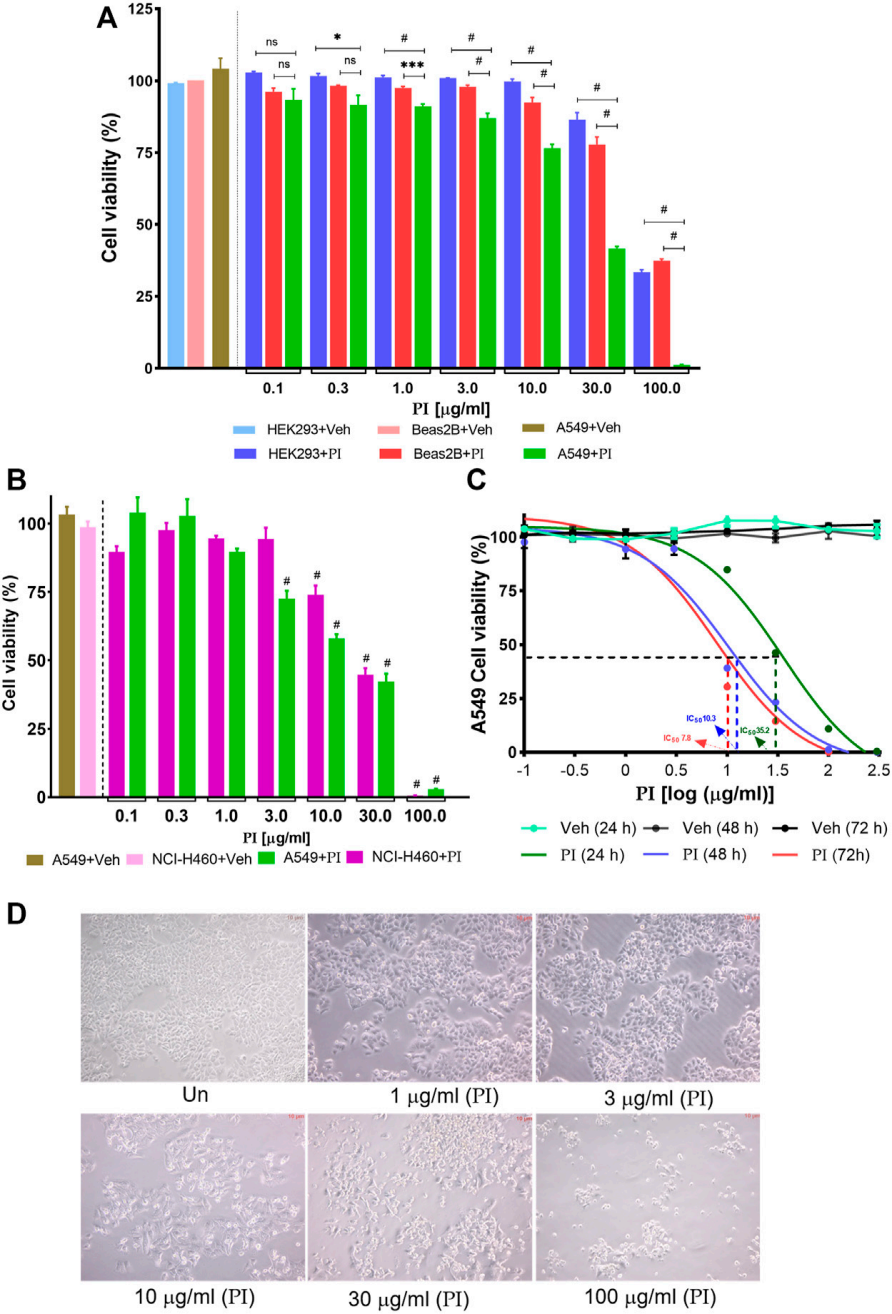
*Pistacia integerrima* induces selective toxicity in non-small cell lung cancer cells. Cytotoxic effects of different PI concentrations post 24 h of treatment were assessed with Alamar blue. Percent cell viability with respect to untreated was calculated in vehicle and treated conditions. DMSO of 0.9% present in 300 μg/ml of PI (3 times of maximum dose) served as vehicle control in all cell viability assays. **(A)** Percent cell viability of PI in HEK293, Beas2B, and A549 cells (*n* = 3). A significant difference was evaluated in A549 with respect to HEK and Beas2B percent cell viability. **(B)** Percent cell viability in NCI-H460 and A549 cells (*n* = 3). A significant difference was evaluated in A549 and NCI-H460 with respect to their respective vehicle control. Two-way ANOVA employing Tukey’s multiple comparison test (*α* = 0.05) was used to analyze significant difference in the cell viability of treated cells. Error bars represent mean ± SEM; significance of data was represented as *p < 0.05, ***p < 0.0005, **#** *p* < 0.0001 and not significant (ns) if *p* > 0.05. **(C)** A549 cells were treated with PI for 24, 48, and 72 h. The log(μg/ml) concentrations −1, −0.5, 0, 1, 1.5, 2, and 2.5 correspond to 0.1, 0.3, 1, 3, 10, 30, 100, and 300 μg/ml of PI (*n* = 3). The half maximal inhibitory concentration (IC_50_) of PI was evaluated by fitting the non-linear regression dose–response curve on PI-treated curves in GraphPad 8.0. Broken lines (---) mark the IC_50_ on the non-linear fit. **(D)**. Brightfield images of A549 cells post 24 h of PI treatment at indicated concentrations. Images were captured under the inverted microscope at 40 × magnification.

To address the effect of PI on the viability of cancer cells, non-small cell lung cancer cells, A549, and non-cancerous cell lines, the human embryonic kidney cells HEK293, and human bronchial epithelial cells, Beas2B, were evaluated. Doses of PI ranging from 0.3 μg/ml to 10 μg/ml did not affect the viability of HEK293 and Beas2B, which on the contrary were comparatively more cytotoxic to A549 cells. PI at 30 μg/ml depreciated the cell viability of 10% HEK293 and 20% Beas2B cells in comparison to 60% in A549 cells. However, PI concentration of 100 μg/ml compromised the survival of all cell types irrespective of being from non-cancer or cancer lineage (Figure 1A). The specificity of PI toward cancer cells was also probed in NCI-H460 cells, another non-small cell lung cancer cell type. Although PI induced comparable cytotoxicity in both cell types, A549 and NCI-H460, 3 μg/ml of PI caused a significant reduction in cell viability of A549 (Figure 1B). A comparative difference of PI toward A549 and NCI-H460 is also evident at 10 μg/ml concentration but collectively the data suggest that PI is preferentially more cytotoxic to lung cancer cells (Figures 1A,B). The half-maximal inhibitory concentration (IC_50_) of PI in A549 post 24, 48, and 72 h of treatment concomitantly reduced from 35.2 μg/ml to 10.3 μg/ml and 7.8 μg/ml, respectively (Figure 1C). Brightfield microscopic images indicate cells turning into spherical cellular bodies, undergoing shrinkage, and subsequently reducing in numbers suggesting that A549 cells confront morphological changes with PI (Figure 1D) (Elmore, 2007).

### *Pistacia integerrima* Inhibits Clonogenic and Migration Potential of Lung Cancer Cells

**FIGURE 2:**
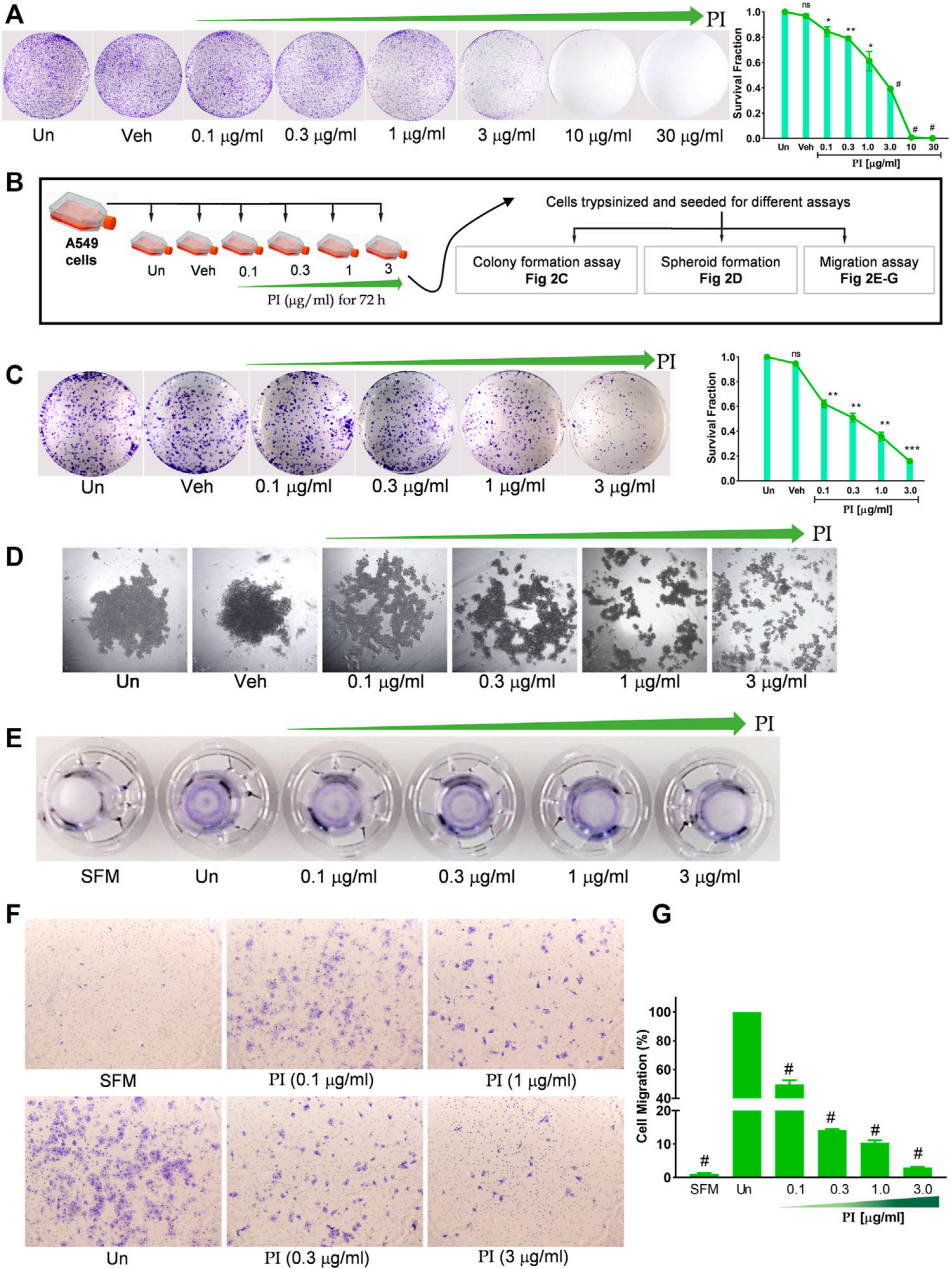
*Pistacia integerrima* inhibits clonogenic and migration potential of lung cancer cells. **(A)** A549 cells seeded in six-well tissue culture plate at 5000 cells/well density, treated with indicated PI concentrations for 72 h. Cells were stained with 0.2% crystal violet and imaged using a digital camera. Colonies were counted and expressed in survival fraction (right panel) (*n* = 3). **(B)** Illustration depicting experimental approach followed in **(C–G)**. **(C)** A549 cells seeded at 1000 cells per well in a six-well tissue culture plate, allowed to grow for 10 days under cell culture conditions with media replacement every third day. Cells were stained with 0.2% crystal violet and imaged using a digital camera. Colonies were counted to calculate survival fraction (*n* = 3). **(D)** Spheroid-like structures were allowed to grow with A549 cells through the hanging drop method for 72 h. Structures were imaged under an inverted microscope. **(E)** Images of the transwell inserts (inverted) captured using the digital camera show the comparative intensity of crystal violet indicating migration of A549 cells. **(F)** Representative images captured under the microscope showing the migrated cells in trans-well. **(G)** Number of cells migrated in response to PI treatment were counted and graphically represented. Percentage of migrated cells was counted with respect to untreated. One-way ANOVA was used to analyze significant differences with respect to untreated conditions. Error bars represent mean ± SEM; significance of data represented as *p < 0.05, **p < 0.005, ***p < 0.0005, ^**#**^ *p* < 0.0001 and not significant (ns) if *p* > 0.05.

The colony-forming potential of A549 was assessed under the influence of PI. Cells seeded at low density when treated with PI for 72 h, formed a less number of colonies than vehicle or untreated. A significant dose-dependent reduction in the survival fraction, indeed negligible survival at PI concentrations, 10 μg/ml and 30 μg/ml, indicates a challenge for A549 cells to maintain viability at low density in the presence of PI (Figure 2A). The assay also suggests altered proliferation rates in A549 with PI, albeit qualitatively. To validate the potential of PI in inhibiting the colony formation or metastatic characteristics of A549, cells were treated with non-lethal doses, i.e., 0.1 μg/ml, 0.3 μg/ml, 1 μg/ml, and 3 μg/ml for 72 h and re-seeded them at an equal density for different assays. Notably, after re-seeding, PI was not added to cells during the time course of these assays (Figure 2B). The number of colonies and corresponding survival fraction significantly reduced in cells that were pre-treated with PI compared to vehicle or untreated. We observed ∼20% reduction in the number of colony formation potential of cells that were pre-treated with 0.1 μg/ml and 0.3 μg/ml of PI. The survival fraction that reflects the colony formation potential was reduced to 0.6 to 0.8 at 1 μg/ml and 3 μg/ml treatment of PI, respectively (Figure 2C). A549 cells were subsequently allowed to develop tumor spheroid-like structures for 6 days. Untreated and vehicle showed a 3D spheroid-like formation, but the cells that were pre-treated with PI showed dispersed A549 cells suggesting the inability of these cells to form discrete tumor-like spheroid (Figure 2D). To associate that PI modulates the metastatic behavior of A549 cells, we performed transwell migration assay. The crystal violet staining of the inserts depicts lesser migration of cells that were pre-treated with PI (Figure 2E). The number of cells migrated was counted under the brightfield microscope. Cells pre-treated showed a dose-dependent reduction in migration in comparison to untreated cells (Figures 2E–G). Collectively, we conclude that PI inhibits the colony formation, tumor-like developments, and migration potential of A549 cells. Notably, the concentrations of PI tested in this experiment have no effect on the cell vitality but efficiently mitigate the cancerous characteristics of A549 cells.

### *Pistacia integerrima* Potentiates ROS-Independent Apoptosis in Lung Cancer Cells

**FIGURE 3:**
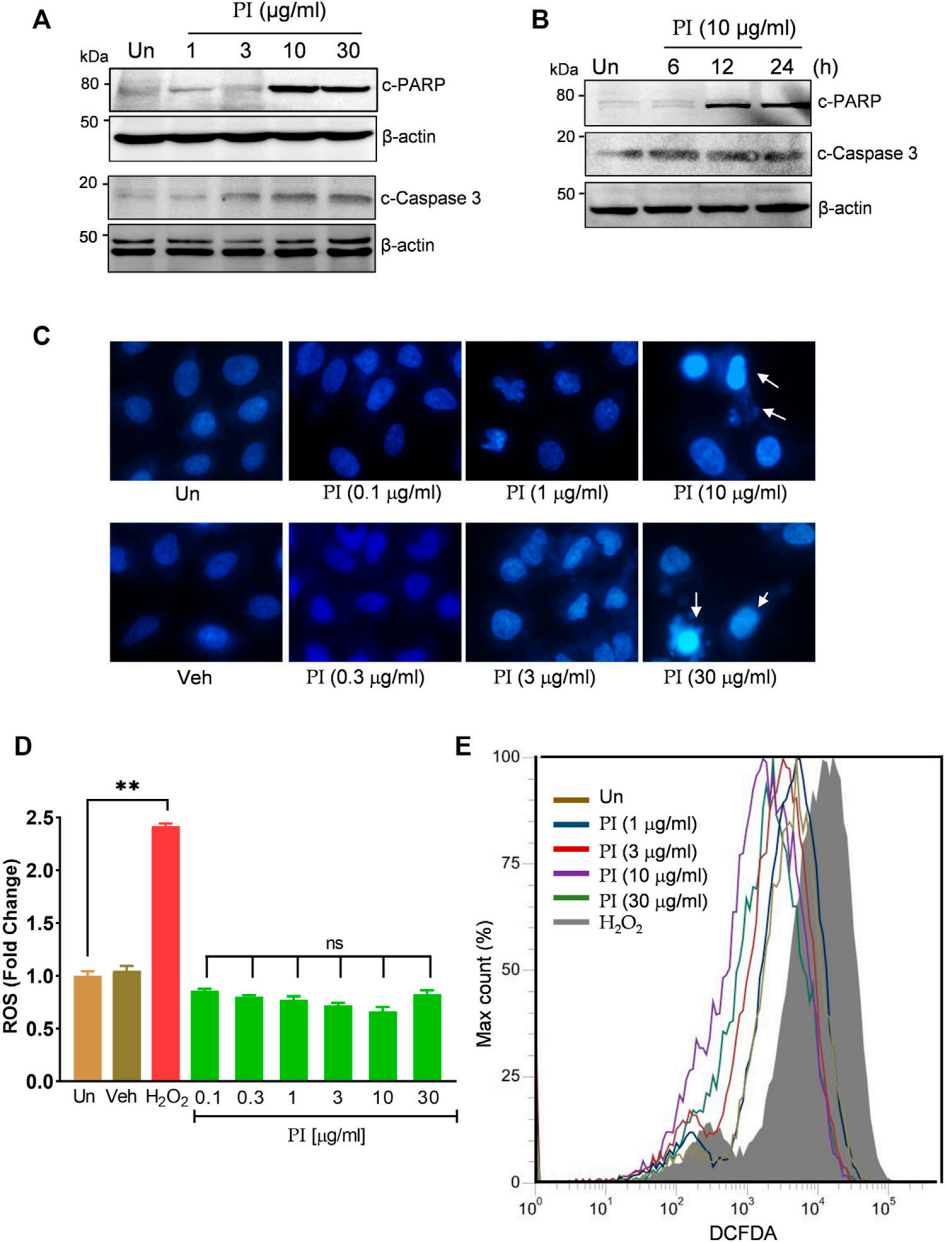
*Pistacia integerrima* potentiates ROS-independent apoptosis in lung cancer cells. **(A)** Whole cell lysates of A549 cells post 24 h of treatment at indicated doses were subjected to Western blotting using antibodies against cleaved PARP1 and cleaved caspase 3. *β*-Actin served as the loading control. **(B)** Whole cell lysates of A549 cells treated at 10 μg/ml of PI for 6, 12, and 24 h time period was subjected to Western blotting using antibodies against cleaved PARP1 and cleaved caspase 3. *β*-Actin served as a loading control. **(C)** A549 cells treated at indicated doses of PI for 24 h were stained with DAPI and imaged under a fluorescent microscope. **(D)** A549 cells post PI treatment for 24 h were stained with DCFDA for 45 min. Fluorescent units were measured using a multi-plate reader and were expressed in relative fold change with respect to untreated. The fold change difference in PI treatments was not-significant (ns) with respect to untreated. **(E)** DCFDA fluorescence expressed as percent max count was determined in A549 cells with indicated treatments for 24 h using a flow cytometer. H_2_O_2_ served as a positive control for DCFDA fluorescence. Error bars represent mean ± SEM; significance of data represented as **p < 0.005 and **#** *p* < 0.0001 and not significant (ns) if *p* > 0.05.

In order to determine the underlying mechanism of PI cytotoxicity toward lung cancer cells, we assessed the apoptotic markers, cleaved PARP1 and cleaved caspase 3. PI dose dependently increased the cleaved PARP1 and cleaved caspase 3 levels at 10 μg/ml and 30 μg/ml (Figure 3A). At 10 μg/ml, a time-dependent impact of PI was observed on cleaved PARP1 and cleaved caspase 3 levels (Figure 3B). To assess the morphological changes in the nuclear bodies of A549 cells with PI, DAPI staining was performed. A549 nuclei showed chromatin condensation, membrane blebbing, and formation of apoptotic bodies at 10 μg/ml and 30 μg/ml of PI, whereas at 1 μg/ml and 3 μg/ml it could only induce mild nuclear shrinkage and morphological changes (Figure 3C). Since oxidative stress is one of the metabolic factors striding cells toward apoptosis, we also assessed intracellular ROS levels. Non-significant change in the ROS levels of A549 cells with PI treatment was observed (Figure 3D). Similarly, in the flow cytometry experiments, peaks indicating DCFDA fluorescent counts were comparable in untreated and PI-treated A549 cells; on the contrary, an evident right shift of peak (grey) emerged with H_2_O_2_ treatment (Figure 3E). Taken together, the data indicate that PI-mediated reduction in cell viability is largely due to apoptosis but indulges mechanisms that are independent of ROS. Furthermore, to decipher the mechanism of cell death through PI, we looked into the probable role of PI-mediated autophagy in lung cancer cells, as excessive autophagy has been earlier linked with apoptosis in cancer cells (Lenardo et al., 2009).

### *Pistacia integerrima* Induces AMPK-Dependent Autophagic Cell Death in Lung Cancer Cells

**FIGURE 4:**
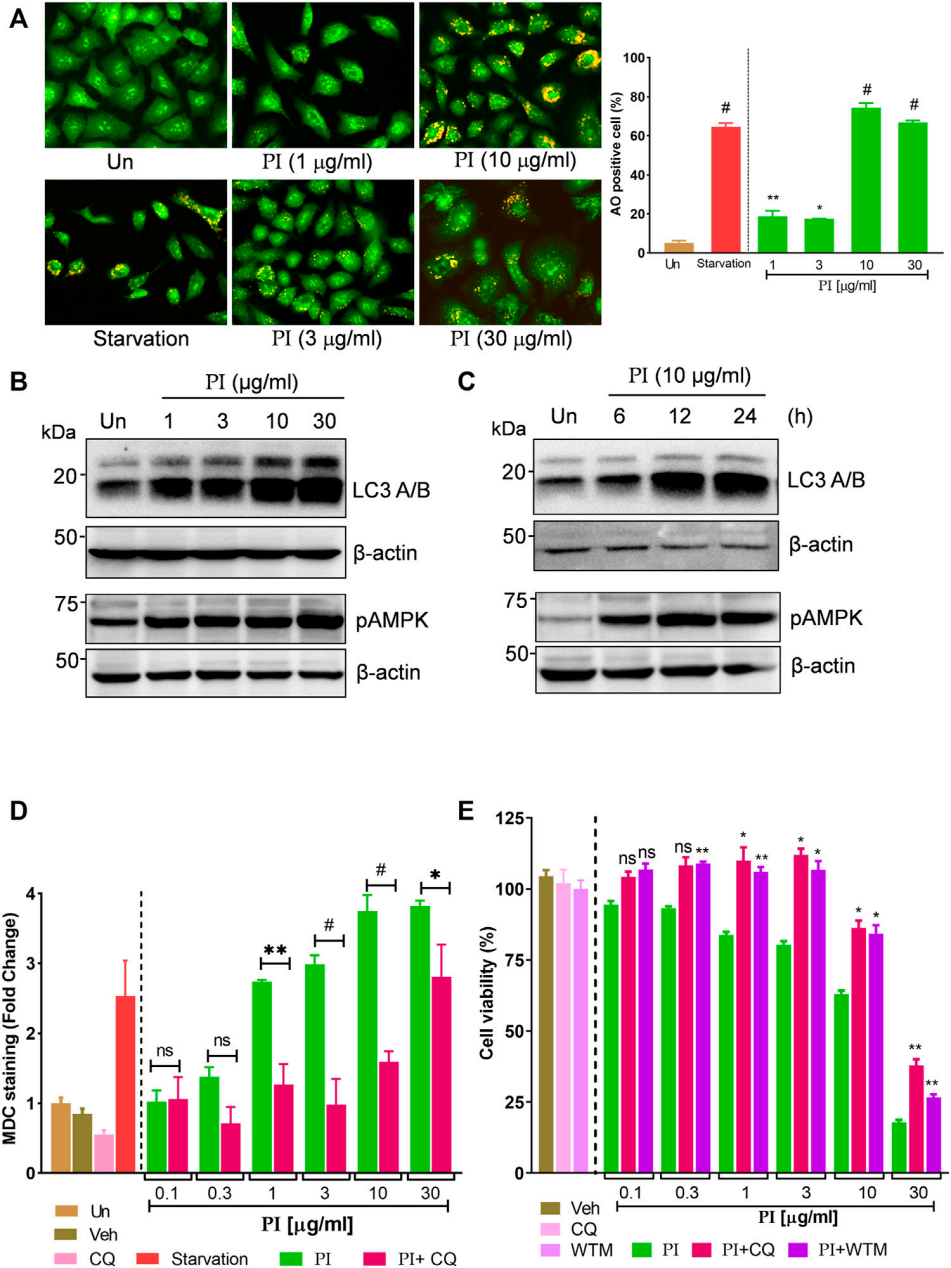
*Pistacia integerrima* induces AMPK-dependent autophagic cell death in lung cancer cells. **(A)** Acridine orange staining of A549 cells post 24 h treatment with PI at indicated doses. Each image is zoomed in to clearly show the acidic vacuoles. The right panel shows the percentage of cells positive for acidic vesicular organelles (AO) in each treatment. Significance is evaluated with respect to untreated. **(B)** Whole cell lysates of A549 cells, post 24 h of treatment at indicated doses, were subjected to Western blotting using antibodies against LC3 A/B and pAMPK. *β*-Actin served as a loading control. **(B)** Whole cell lysates of A549 cells treated at 10 μg/ml of PI for 6, 12, and 24 h time periods were subjected to Western blotting using antibodies against LC3 A/B and pAMPK. *β*-Actin served as the loading control. **(D)** A549 cells treated with PI alone or together with chloroquine (CQ) for 24 h were stained with MDC. Fluorescence was expressed as fold change with respect to untreated. A significant difference in fold change of MDC staining is calculated in PI + CQ treatment with respect to PI. **(E)** Cell viability with Alamar blue was assessed in A549 cells treated with indicated treatments for 24 h. Cells were treated with chloroquine (CQ) at 10 μM and wortmannin (WTM) at 500 nM concentration. Significance in PI + CQ and PI + WTM is calculated with respect to PI alone. Error bars represent mean ± SEM; significance of data represented as *p < 0.05, **p < 0.005, ***p < 0.0005, and ^**#**^ *p* < 0.0001 and not significant (ns) if *p* > 0.05.

Autophagy is characterized by the increased levels of acidic vesicular organelles in the cell cytoplasm. Acridine orange is a green fluorophore fluoresce red in acidic vesicular organelles (Thomé et al., 2016). The merged images of green and red fluorescence show a significant increase in the yellow foci with an increasing concentration of PI (Figure 4A) indicating induction of autophagy. More than 60% of cells were positive for yellow foci under starvation (positive control) and PI treatment at 10 μg/ml and 30 μg/ml (Figure 4A, right panel). PI-driven autophagy was ascertained by a robust increase in the hallmarks of autophagy, pAMPK (phospho-AMPK at Thr 172), and phosphatidylethanolamine-conjugated LC3 (LC3 II), in a dose- (Figure 4B) and time- (Figure 4C) dependent manner. We further substantiated these findings with monodansylcadaverine (MDC) that stains autophagic vacuoles in the cells. MDC staining showed a thrust of 1.5-to-3.5-fold in PI-treated cells which then significantly reduced in the presence of autophagy inhibitor, chloroquine (CQ) (Figure 4D). To test whether PI-induced autophagy guides cancer cells toward cell death, we performed a cell viability assay in presence of autophagy inhibitors, chloroquine (CQ) and wortmannin (WTM). PI showed dose-dependent cytotoxicity as shown earlier, but when coupled with CQ or WTM, the cytotoxicity of PI was significantly reduced. Autophagy inhibitors, CQ and WTM, rescued around 25–30% of cells from cytotoxic cell death induced by PI treatment (Figure 4E). Taken together, these data suggest PI induces autophagic cell death in A549 lung cancer cells.

### Bioactivity-Guided Fractionation of *Pistacia integerrima* Identified Penta-O-Galloyl-*β*-D-Glucose as a Bioactive Phytocompound

**FIGURE 5:**
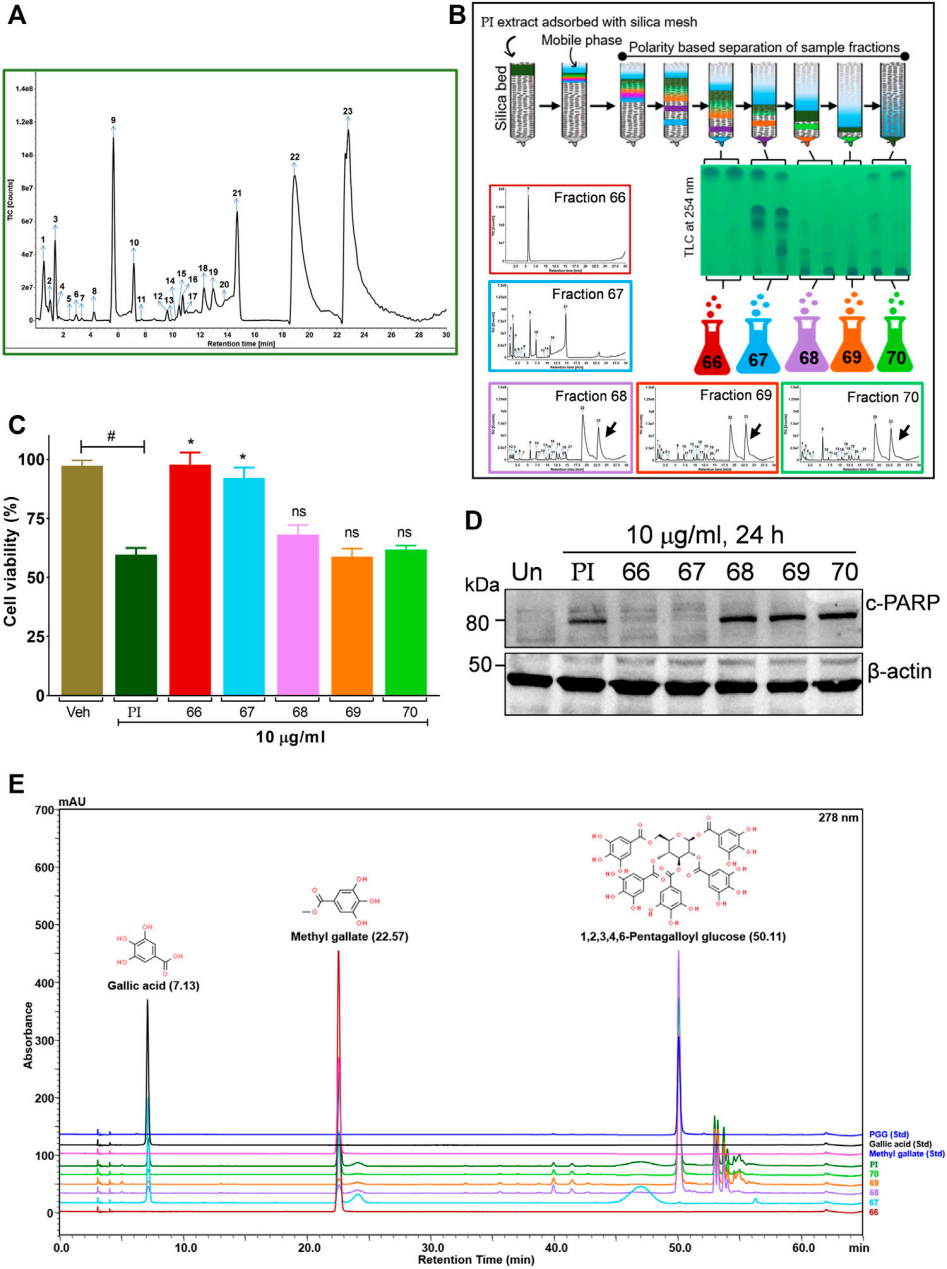
Bioactivity-guided fractionation of *Pistacia integerrima* identified penta-O-galloyl-*β*-D-glucose (PGG) as a bioactive phytocompound**. (A)** Chromatogram of total ion count (TIC) versus retention time (min) shows peaks labeled with arrows and numbers. Peaks representing the phytometabolite were identified by UPLC/QToF-MS and detailed in Table 1. **(B)** Schematic showing fractionation of PI extract through column chromatography. A total of 34 aliquots were collected as fractions 66,67,68, 69, and 70. UPLC/QToF-MS generated the chromatograms for all the fractions. Peaks 22 and 23 (arrow labeled) corresponding to 1,2,3,4,6-Pentagalloyl Glucose (PGG) and 2-O-digalloyl-1,3,4,6-tetra-O-β-D-galloyl Glucose were not detected in fraction 66 and 67. **(C)** Percent cell viability was evaluated in A549 cells treated with different fractions at 10 μg/ml for 24 h. Alamar blue assay determined the percent viability in all treatments (*n* = 4). Significance in percent viability in all treatments is calculated with respect to PI. Error bars represent mean ± SEM; significance of data represented as *p < 0.05 and not significant (ns) if *p* > 0.05. **(D)** Whole cell lysates of A549 cells, post 24 h of treatments at 10 μg/ml, were subjected to Western blotting using antibodies against cleaved PARP1. *β*-Actin served as a loading control. **(E)** Overlayed chromatogram generated through UHPLC showing the absorbance and retention time of standards, fractions, and PI.

To further gain insights into the phytometabolites responsible for the observed anticancer activity of PI, we performed UPLC/QToF-MS and identified 23 phytometabolites. Analysis of peaks in the TIC chromatogram (Figure 5A) showed that PI extract is majorly comprised of a range of gallotannins including gallic acid, methyl gallate, and penta-O-galloyl-*β*-D-glucose (PGG) along with other galloyl glucose forms (Table 1). To address the bioactive phytocompounds that impart the anticancer activity in PI, the complexity of the extract was reduced through a fractionation strategy. Hydro-methanolic extract of PI was passed through the silica bed using different ratios of chloroform and methanol as the mobile phase. In total, 34 flow-through aliquots were collected and tested for their TLC profile. On the basis of similar banding patterns, aliquots were pooled into five individual fractions, namely, 66, 67, 68, 69, and 70 (Figure 5B). These collected fractions were then subjected to UPLC/QToF-MS to generate a comparative TIC chromatogram. Remarkably, a specific peak corresponding to penta-O-galloyl-*β*-D-glucose was not detected in 66 and 67 (Figure 5B). The differential phytometabolic profile of the fractions possessing distinct biological activity was subsequently determined. Notably, fractions 66 and 67 showed a non-significant reduction in cell viability, indeed comparable to untreated cells. On the contrary, fractions 68, 69, and 70 showed 35–40% cytotoxicity much in line with unfractionated PI.

**TABLE 1:** List of phytometabolites identified in hydro-methanolic extract of *Pistacia integerrima* through UPLC/QToF MS.

S.N.^a^	Component name	Formula	Neutral mass (Da)	Observed m/z	RT (min)	Response	Adduct
1	3-Galloyl-glucose	C_13_H_16_O_10_	332.0744	331.0673	0.61	557,725	-H
2	1-Galloyl-glucose	C_13_H_16_O_10_	332.0744	331.0670	1.05	662,680	-H
3	Gallic acid	C_7_H_6_O_5_	170.0215	169.0142	1.41	1,499,659	-H
4	6-O-Galloyl glucose	C_13_H_16_O_10_	332.0744	331.0672	1.50	161,502	-H
5	Ethyl gallate	C_9_H_10_O_5_	198.0528	243.0511	2.49	74,510	+HCOO
6	3-Galloylshikimic acid	C_14_H_14_O_9_	326.0638	325.0567	2.95	303,895	-H
7	4-O-Galloylshikimic acid	C_14_H_14_O_9_	326.0638	325.0567	3.36	138,736	-H
8	2-(Hydroxymethyl)phenyl hexopyranoside	C_13_H_18_O_7_	286.1053	331.1036	4.25	313,570	+HCOO, -H
9	Methyl gallate	C_8_H_8_O_5_	184.0372	183.0298	5.68	4,727,517	-H
10	Digallic acid	C_14_H_10_O_9_	322.0325	321.0257	7.17	1,877,370	-H
11	2,4,6-Tri-O-galloyl-*β*-D-glucose	C_27_H_24_O_18_	636.0963	635.0891	7.74	120,037	-H, 2x (-H)
12	1,2,3-O-Tri-galloyl-beta-d-glucose	C_27_H_24_O_18_	636.0963	635.0903	9.61	427,250	-H, 2x (-H)
13	1,2,6-Tri-O-galloyl-b-D-glucose	C_27_H_24_O_18_	636.0963	635.0904	10.03	129,110	-H, 2x (-H)
14	6′-O-Galloylsalicin	C_20_H_22_O_11_	438.1162	437.1102	10.28	227,787	-H
15	1,2,4,6-Tetragalloyl glucose	C_34_H_28_O_22_	788.1072	787.1027	10.47	563,628	-H, 2x (-H)
16	2,6-Dihydroxy-4-(methoxycarbonyl)phenyl 3,4,5-trihydroxybenzoate	C_15_H_12_O_9_	336.0481	335.0412	10.74	1,008,912	-H
17	2,3,4,6-Tetragalloyl glucose	C_34_H_28_O_22_	788.1072	787.1008	10.98	153,333	-H, 2x (-H)
18	1,2,3,6-Tetra-O-galloyl-*β*-D-glucose	C_34_H_28_O_22_	788.1072	787.1016	12.29	1,171,529	-H, 2x (-H)
19	1,2,3,4-Tetragalloyl glucose	C_34_H_28_O_22_	788.1072	787.1020	12.98	1,782,315	-H, 2x (-H)
20	1,3,4,6-Tetragalloyl glucose	C_34_H_28_O_22_	788.1072	787.1012	13.82	215,321	-H, 2x (-H)
21	2,3-Dihydroxy-5-(methoxycarbonyl)phenyl 3,4,5-trihydroxybenzoate	C_15_H_12_O_9_	336.0481	335.0410	14.73	6,366,418	-H
22	1,2,3,4,6-Pentagalloyl glucose	C_41_H_32_O_26_	940.1182	939.1135	18.85	25,421,378	-H, 2x (-H)
23	2-O-digalloyl-1,3,4,6-tetra-O-*β*-D-galloyl glucose	C_48_H_36_O_30_	1092.129	1091.128	22.86	8,232,115	-H

a S.N. represents the peak number depicted in the chromatogram (Figure 5A).

To gain a deeper mechanistic understanding, cleaved PARP1 levels were tested in A549 cells independently treated with PI fractions. Interestingly, cells treated with fractions 66 and 67 showed negligible levels of cleaved PARP1, whereas significant levels were determined in cells treated with PI, 68, 69, or 70 (Figure 5D). Overlayed chromatograms indicated gallic acid, methyl gallate, and penta-O-galloyl-*β*-D-glucose (PGG), the important gallotannins which were quantitated in all the fractions, in addition to PI, through UHPLC (Figure 5E). PI fractions 68, 69, and 70 contain 19, 35, 24, and 27% (w/w) of PGG, respectively, whereas PGG remained undetected in fractions 66 and 67 in line with UPLC/QToF-MS (Figure 5E; Table 2). Hence, the data suggest that the anticancer properties of PI are attributed primarily to PGG and not gallic acid or methyl gallate.

**TABLE 2:** UHPLC analysis of PI and its fractions for the phytometabolite contents.

S.N.	Sample name	(% w/w)		
		PGG	Gallic acid	Methyl gallate
1	PI extract	19.12	3.89	15.51
2	Fraction 66	0	0	99.68
3	Fraction 67	0	15.29	12.26
4	Fraction 68	34.61	0.91	1.30
5	Fraction 69	24.42	0.43	0.97
6	Fraction 70	27.41	0.60	6.50

### Penta-O-Galloyl-*β*-D-Glucose Activates AMPK and ERK and Inhibits STAT3 Signaling in Lung Cancer Cells

**FIGURE 6:**
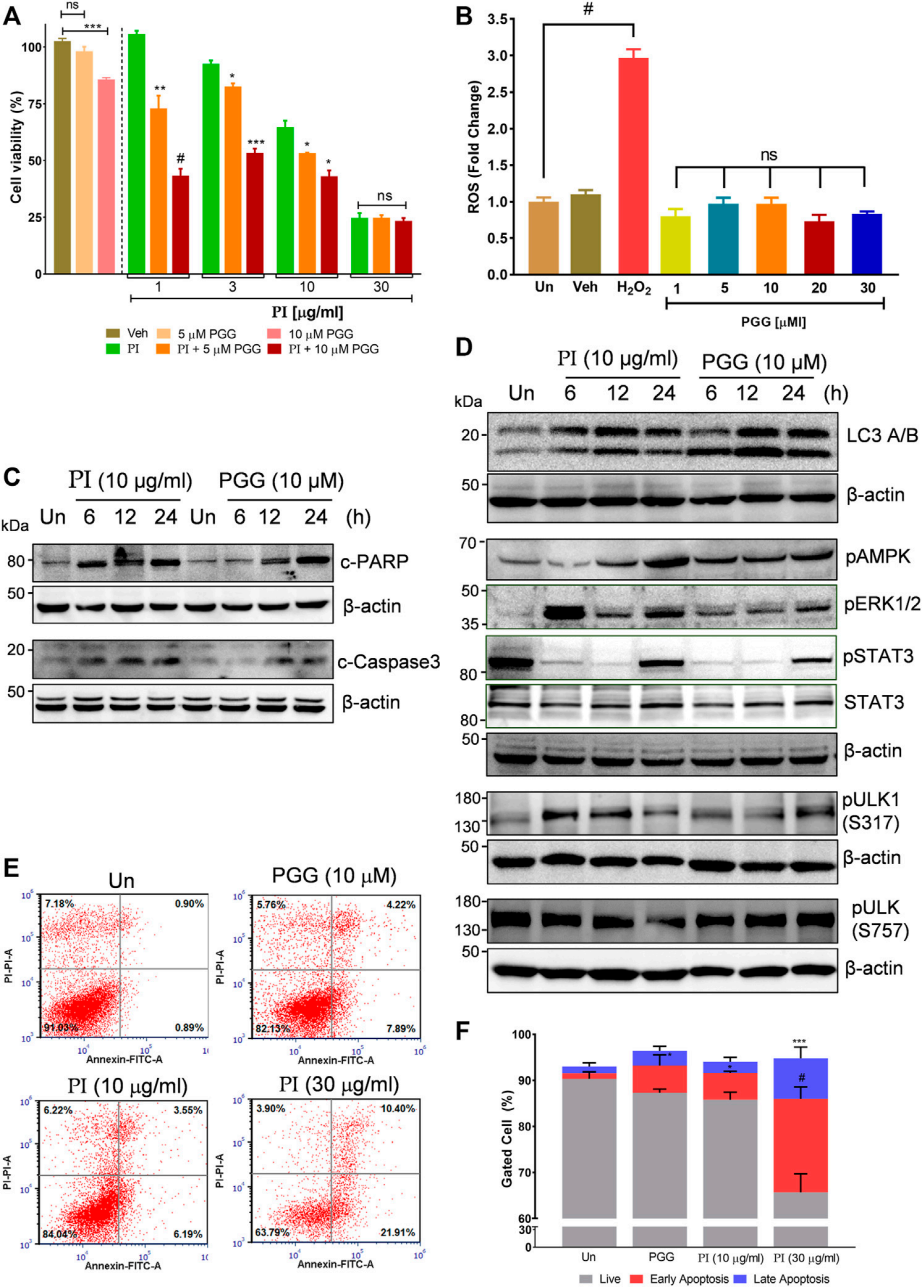
Penta-O-galloyl-*β*-D-glucose (PGG) activates AMPK and ERK and inhibits STAT3 signaling in lung cancer cells. **(A)** Percent cell viability calculated in A549 cells treated for 24 h with 5 and 10 μM of PGG alone or in presence of PI at indicated concentrations. Significance in PI + 5 μM PGG and PI + 10 μM PGG was calculated with respect to PI alone (*n* = 3). Error bars represent mean ± SEM; significance of data represented as *p < 0.05, **p < 0.005, ***p < 0.0005, and ^#^ *p* < 0.0001 and not significant (ns) if *p* > 0.05. **(B)** A549 cells post PGG treatment for 24 h were stained with DCFDA for 45 min. Fluorescent units were measured using a multi-plate reader and were expressed in relative fold change with respect to untreated. The fold change difference in PI treatments was non-significant (ns) with respect to untreated. H_2_O_2_ served as a positive control for DCFDA fluorescence. Error bars represent mean ± SEM; significance of data represented as ^#^ *p* < 0.0001 and not significant (ns) if *p* > 0.05. **(C)** Whole cell lysates of A549 cells, at indicated treatments of PI (10 μg/ml) and PGG (10 μM), were subjected to Western blotting using antibodies against cleaved PARP1 and cleaved caspase 3. *β*-Actin served as the loading control. **(D)** Whole cell lysates of A549 cells, treated with PI (10 μg/ml) and PGG (10 μM), were subjected to Western blotting using antibodies against LC3A/B, pAMPK, pERK1/2, pSTAT3(Y705), STAT3, pULK1(S317), and pULK1(S757). *β*-Actin served as a loading control. **(E)** Representative dot plots obtained from flow cytometric analysis of A549 cells with indicated treatments of PI and PGG (10 μM) for 24 h. Cells were stained with PI (propidium iodide) and Annexin V FITC to evaluate the percentage of cells undergoing apoptosis, indicated in each quadrant. **(F)** The panel shows graphically the percentage of live and apoptotic cells representing mean ± SEM from three experimental replicates.

The aforementioned results collectively indicate that the phytomolecule, PGG, is an important determinant in PI-mediated autophagic cell death. In order to further validate, we performed experiments using commercially available pure PGG to compare the PI-induced changes in the molecular signatures of autophagy and apoptosis. First, to ascertain the role of PGG in PI-mediated cancer cell cytotoxicity, we spiked PI with pure PGG and tested it on A549 cells. Interestingly, PGG-spiked PI significantly reduced the cell viability up to ∼65%, even at the sub-lethal doses of PI (1 μg/ml and 3 μg/ml) (Figure 6A), indicating PGG is an important determinant of PI-mediated cytotoxicity. Notably, PGG alone induced 5 and 20% cytotoxicity in A549 cells at 5 and 10 μM concentrations, respectively. The cytotoxicity mediated through PGG is also independent of oxidative stress, very much like PI, in A549 cells (Figure 6B). We further deciphered through immunoblotting, if the observed changes in the molecular signatures by PI are driven by PGG. Both PI and PGG treatment upregulated cleaved PARP1 and cleaved caspase 3 in A549 cells indicating similarity in apoptosis that is mediated through caspase 3. However, PI treatment showed early and higher signs of both apoptotic markers (Figure 6C). PGG acts similar to PI in upregulating LC3 II and pAMPK levels indicating that PGG present in PI directs the AMPK-driven autophagy in lung cancer cells (Figure 6D). We further tested the levels of ULK1 phosphorylation at Ser317 and Ser757 through Western blotting. It is previously established that AMPK activation triggers autophagy by phosphorylating ULK1 at Ser317. On the other hand, mTOR activation inhibits autophagy by phosphorylating ULK1 at Ser757 to disrupt the ULK1 and AMPK interaction. Autophagy has earlier been associated with ERK activation and STAT3 inhibition. The two have been shown to target STAT3 activation, and PI and PGG treatment in A549 cells activates ERK but alleviates pSTAT3 in comparison to stable STAT3 levels at 6 and 12 h. Although pSTAT3 levels appear to be restored at 24 h, it is still less than the untreated condition (Figure 6D). The modulations in the two crucial signaling axes lead to apoptosis in 12% of cells upon PGG treatment, while a 9–32% increase in cellular apoptosis was observed in dose-dependent PI treatment (Figures 6E,F). Collectively, the data suggest that PGG and PI share a similar mode of action, and PGG governs the PI activity that could impede lung cancer progression.

## Discussion

Historically, plants have remained an important ingredient in medicinal preparations and gradually even modern science is recognizing the biological activity in their metabolites. A detailed report on medicines approved by the US Food and Drug Administration (FDA) has accentuated the importance of small molecules obtained from natural sources. Around 34% of approved medicines between 1981 and 2010 were developed from small molecules derived from natural products (Newman and Cragg, 2020).

The present study aims to identify a naturally occurring phytocompound that has the potential to be built into a chemotherapeutic against lung cancer. We re-examined the documented medicinal values of the gall of *Pistacia integerrima* and deciphered its role in human non-small cell lung cancer cells, A549. The preliminary data on cytotoxicity indicated specificity in the PI extract to target only lung cancer cells (Figures 1A,B). Moreover, the half-maximal inhibitory concentration of PI post 24 h treatment was 35.2 μg/ml (Figure 1C), which suggests that, unlike other plant extracts that are effective at high milligram concentrations, PI is showing bioactivity at microgram concentration. The maximum dose we incorporated to study the bioactivity of PI is 30 μg/ml. We further explored the concentrations of PI that were not cytotoxic to A549 cells but effectively retarded the cancer attributes in A549 cells. Cells pre-treated with PI at lower concentrations from 0.1 to 3 μg/ml reduced the A549 colony formation potential, inhibited the tumor-forming property, and most importantly impeded the metastatic capability of cancerous cells (Figures 2A–F).

Determining the cytotoxicity of PI in A549 cells, the mode of cell death was ascertained to be apoptosis. A substantial increase in the levels of cleaved PARP and cleaved caspase 3 with a visualized deformation, fragmentation, and condensation of nuclear bodies validates apoptosis in A549 cells post PI treatment (Figures 3A–C) (Elmore, 2007). One of the strategies of targeting cancer cells by various chemotherapeutic agents is to inflate the cellular ROS levels exuberantly that resulting in apoptosis. EGCG, benzyl isothiocyanate, and trichostatin A are a few examples of drugs that follow similar mechanisms (Qanungo et al., 2005; Gahr et al., 2007; Zhang et al., 2008). However, ROS elevation upon PI treatment was absent in A549 cells suggesting a different mechanism of action associated with PI that mediates apoptosis in A549 cells (Figures 3D,E). We did not observe an increase in ROS probably due to the anti-oxidant properties of *Pistacia integerrima*, as previously reported (Eshwarappa et al., 2015). To gain insights into the components of cellular machinery that trigger apoptosis upon PI treatment, we investigated other programmed cell death forms that involve autophagy. Although autophagy is a cell’s protective response to stress, at times upon hyperactivation, this pro-survival mechanism turns into a signal for cell death (Mizushima et al., 2010; Shimizu et al., 2014; Linder and Kögel, 2019). We demonstrate the occurrence of autophagy through acridine orange staining, activation of AMPK, and increasing levels of LC3 II (Figures 4A–C). MDC staining showed that CQ inhibits PI-mediated autophagy (Figure 4D). Most importantly, an increase in the percentage of cell viability even in the presence of PI but coupled with autophagy inhibitors, CQ and WTM, indicates a concurrence of PI-mediated “autophagic cell death” (Figure 4E) (Mizushima et al., 2010; Mauthe et al., 2018). Since PI-mediated apoptosis was not mediated through oxidative stress, non-selective autophagic cell death may be occurring with PI treatment (Shimizu et al., 2014).

We further investigated the phytometabolite present in PI that curtails the progression and survival of A549 cells. Upon column chromatography, we collected 34 fractions at different time intervals but pooled them into 5 and characterized them using UPLC/QToF MS along with the PI extract. A thorough analysis showed PI as a rich source of gallotannins, the naturally occurring phenolic metabolites, and belongs to the class of hydrolyzable tannins (Ahmad et al., 2010; Machida et al., 2021). We majorly identified gallic acid, methyl gallate, and galloyl glucose containing different galloyl groups (Table 1) in PI extract (Figure 5A). Interestingly, comparative total ion chromatograms generated through UPLC/QToF-MS analysis of fractions and PI extract showed that 66 is distinct from all fractions since it was highly enriched with methyl gallate. In addition, a substantial response peak corresponding to PGG remained undetected in 66 and 67 (Figure 5B). Chromatograms also indicate that we have successfully separated distinct fractions from PI that may have differential biological activity. Hence, we looked into the levels of cell viability in A549 cells in presence of these fractions. Interestingly, fractions 66 and 67 did not show any significant cell death (Figure 5C). Furthermore, the absence of cleaved PARP levels in the corresponding cells substantiates that PGG is the bioactive phytometabolite mediating the anticancer potential of PI (Figure 5D). To further validate the findings, UHPLC quantitated the levels of PGG in the fractions and PI extract (Figure 5E). The fractions, namely, 68, 69, and 70 showed 35, 24, and 27 w/w % of PGG (Table 2), respectively, compared to 19 w/w % in PI extract justifying the comparatively less cleaved PARP levels in PI-treated A549 cell lysates (Figure 5D) and simultaneously validates the apoptosis in A549 cells driven through PGG. Previously, reports have shown that PGG is chemically diverse from gallic acid or methyl gallate and exhibits therapeutic potential against cancer and diabetes (Zhang et al., 2009). Our study demonstrates a systematic identification of PGG in the gall of *Pistacia integerrima* that induces A549 cell death.

A549 cells treated with PI and PGG together showed enhanced cell death compared to PI alone or PGG alone indicating that the activity in PI is majorly the result of PGG that can be complemented by spiking the extract with pure PGG. The data clearly indicate that PGG drives ROS-independent anticancer activity of PI (Figures 6A,B). PGG has been previously shown to act cytoprotective *via* direct and indirect anti-oxidative properties. In our study, we show that PGG-mediated anti-oxidative properties in lung cancer are not cytoprotective, rather divert cells toward autophagic apoptosis. Interestingly, PI and PGG showed comparative mechanisms that include cleaved PARP, cleaved caspase 3, pAMPK, and LC3 II overexpression (Figures 6B,C). AMPK activators are promising therapeutic candidates for lung cancer treatment (Lenardo et al., 2009; Thomé et al., 2016). AMPK activation in lung cancer patients indicates a better prognosis and survival (William et al., 2012). Metformin, a medication used to treat hyperglycemia in type-2 diabetes, is now repurposed in cancer therapy, given it could activate AMPK. Natural products, including resveratrol, quercetin, epigallocatechin-3-gallate, berberine, curcumin, and widdrol, inhibit the growth of numerous cancer types by activating AMPK. There are sufficient reports showing that AMPK activation reprograms the cellular metabolic pathways to suppress the cancer progression by regulating crucial players of the cell cycle, autophagy, and apoptosis (Elmore, 2007; Lenardo et al., 2009). PGG being an APMK activator can further be explored for modulations in glycolytic pathways, cell cycle proteins, fatty acid, and cholesterol synthesis. Our study determined that PGG triggers AMPK-ULK1-specific autophagic cell death, but metabolomic changes could also be deciphered further. PGG and PI induce AMPK-dependent autophagic apoptosis independent of oxidative stress. ROS-mediated ERK activation has been frequently mentioned in the literature to induce autophagy and apoptosis, but, on the contrary, we observed PGG and PI-induced ERK activation even in the absence of oxidative stress (Figure 6D) (Cagnol and Chambard, 2010; Hsu et al., 2013). This indicates that PGG and PI employ novel and multiple mechanisms for targeting lung cancer. A549 cells also exhibit KRAS mutations that induce constitutive activation of the downstream signaling cascade leading to cancer cell proliferation, growth, and survival. PGG might have modulating effects on RAS downstream signaling *via* MEK/ERK/PI3K/AKT/mTOR, all being promising drug targets for treating KRAS mutant NSCLC (Tomasini et al., 2016). We demonstrate ERK activation upon PI/PGG treatment; hence, we anticipate that impeding complete KRAS signaling could be uncertain with PGG alone. However, clubbing PGG with other inhibitors may show synergistic or additive blockage of KRAS-driven tumor progression. Our study advocates systems network pharmacology for further mechanistic exploration of PGG in cancer biology.

The signal transducer and activator of transcription 3 (STAT3) is a key transcription factor regulating normal cell growth and survival. Constitutively activated STAT3 has been associated with the pathogenesis of not only non-small cell lung cancer but in almost 70% of cancer types (Fagard et al., 2013). Previous research has developed a strong correlation between STAT3 activation and cancer development. Hence, various small molecule STAT3 inhibitors are in clinical trials as a part of targeted therapy in cancer (Lee et al., 2019). The AMPK-STAT3 axis has been delineated earlier, albeit not in lung cancer, but in regulating monocyte to macrophage differentiation by alleviating STAT3 phosphorylation (Vasamsetti et al., 2015). Interestingly, we determined that PI and PGG also inhibit the phosphorylation of STAT3 at Y705. A previous report has shown that PGG combats the replication of the rabies virus by inhibiting STAT3 activation (Tu et al., 2019). We also observed PGG inhibiting the STAT3 activation, but PI showing a similar inhibitory response toward STAT3 activation validating our finding. Both PI and PGG in early time points significantly inhibited pSTAT3, but at 24 h, a moderate restoration of pSTAT3 is detected (Figure 6D), opening future avenues to explore PGG-mediated anticancer mechanisms that might be feeding in alternate pathways. With all the mechanistic detail, we believe validating the PI activity in various xenograft models could be the future line of research. ROS-independent ERK activation has encouraged us to dissect the mechanistic details of anticancer properties in PGG. In addition, we believe there is a need to address the range of unexplored gallotannins that could possess promising bioactivities.

Collectively, in this study, we have shown galls of *Pistacia integerrima* as a rich source of natural PGG that induces ROS-independent autophagic cell death through ERK activation and alterations in the AMPK-STAT3 signaling axis to combat the lung cancer progression.

## Conclusion

We propose PGG for future clinical investigations against lung cancer. We report gall extract of *Pistacia integerrima* as a novel source of PGG. Simultaneous comparison of PI with pure PGG at the molecular level showed that the biological activity of the extract was PGG driven. PGG can be exploited as a potential AMPK activator/STAT3 inhibitor in developing adjuvant chemotherapeutics against lung cancer. Our study strongly encourages the research on plant-based molecules that can further be exploited as cost-effective adjuvant therapies with acceptable safety profiles.

## Data Availability

The raw data supporting the conclusion of this article will be made available by the authors, without undue reservation.

## Abbreviations

DCFDA, 2′,7′-dichlorodihydrofluorescein diacetate; MDC, monodansylcadaverine; PGG, penta-O-galloyl-*β*-D-glucose; PI, gall extract of *Pistacia integerrima*; UPLC/QToF-MS, ultra-performance liquid chromatography/quadrupole time-of-flight mass spectrometry; UHPLC, ultra-high-performance liquid chromatography.
